# Establishment and biological characterization of radioresistant colorectal cancer cell lines

**DOI:** 10.1002/2211-5463.70016

**Published:** 2025-03-08

**Authors:** Tian‐Yin Qu, Qing Dai, Jing Leng, Lin Fang, Jing‐Jing Ma, Rui Chen, Che Chen, Peng‐Fei Ran, Wen‐Wen Zhou, Chang Liu, Huang‐Fei Yu

**Affiliations:** ^1^ Department of Oncology The First People's Hospital of Zunyi (The Third Affiliated Hospital of Zunyi Medical University) China; ^2^ Cancer Disease Research Institute The First People's Hospital of Zunyi (The Third Affiliated Hospital of Zunyi Medical University) China; ^3^ Department of Clinical Laboratory The Third Affiliated Hospital of Zunyi Medical University (The First People's Hospital of Zunyi) China

**Keywords:** antioxidant, colorectal cancer, DNA damage repair, radioresistant

## Abstract

Radiotherapy resistance is a major cause of recurrence and metastasis in colorectal cancer (CRC). We established radiotherapy‐resistant cell lines to explore the molecular mechanisms of radiotherapy resistance in CRC. HT29 and HCT116 cells were subjected to repeated irradiation at 2 Gy to establish these lines. CCK‐8 assay, colony formation, and xenograft tumor experiments were used to detect the radiosensitivity of the cells. DNA damage repair proteins, GSH content, and intracellular ROS were also assayed in parental and resistant cells. We successfully established HT29R and HCT116R radioresistant cell lines after fractionated irradiation, and the cells showed significant tolerance to further irradiation compared with the parental cells and a stronger capacity for DNA damage repair. Meanwhile, ionizing radiation significantly reduced GSH in HT29 and HCT116 parental cells but had no effect on GSH content in resistant cells. These results demonstrate that radioresistant colorectal cancer cell lines were successfully established by the method of continuous irradiation with 2 Gy. This provides a basis for further exploration of the mechanism of colorectal cancer radiotherapy resistance.

## Graphical abstract

Under ionizing radiation exposure, radiation‐sensitive cancer cells exhibit oxidative stress and DNA damage, while radiation‐resistant cancer cells exhibit strong antioxidant properties and DNA damage repair.

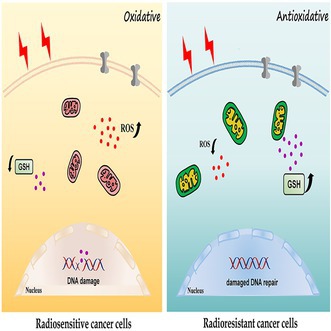

**Abbreviations**
4‐HNE: 4‐Hydroxynonenal
CRC: colorectal cancer
DHE: dihydroethidium
GSH: glutathione
ROS: reactive oxygen species
γH2AX: H2A histone family member X

Colorectal cancer (CRC) is one of the most common malignant tumors of the digestive tract. In 2020 alone, it was responsible for 940 000 cancer‐related deaths worldwide [1]. The incidence of CRC is rising in China. According to the latest data from the National Cancer Centre, CRC ranks as the second most common cancer and has a mortality rate in the top five among urban populations of both men and women, posing a serious threat to public health [2]. Radiotherapy is a major treatment modality and plays a critical role in the comprehensive treatment of CRC. For locally advanced rectal cancer, both preoperative and postoperative radiotherapy hold very important positions [3, 4]. Clinical data show that radiotherapy is effective in achieving safe tumor margins, enhancing anal preservation in patients with low rectal cancer, and reducing surgical complications [5, 6]. For patients with confirmed lymph node metastasis after surgery, postoperative adjuvant radiation is key to preventing local recurrence and regional lymph node metastasis [7, 8]. Meanwhile, palliative radiotherapy remains an indispensable and important tool to prevent obstruction and control clinical symptoms such as pain and bleeding [9].

During radiotherapy, high‐energy X‐rays pass through tumor tissue, causing DNA damage in cancer cells, which directly leads to cell death [10]. Ionizing radiation (IR) also cleaves intracellular water molecules into free radicals (O_2_^·^ and OH^·^) that cause oxidative damage to nucleic acids, lipids, and proteins, contributing to the indirect killing effect [11]. In theory, high‐energy rays used in radiotherapy can accelerate cancer cell death through DNA damage, lipid peroxidation, and the formation of oxygen‐free radicals. However, a large number of clinical studies have found that radiotherapy resistance often develops after a certain radiation dose, limiting the treatment's effectiveness and leading to subsequent recurrence and metastasis [12, 13]. Factors such as cell cycle arrest [14], DNA damage repair [10], the hypoxic tumor microenvironment [15], and the acquisition of stem cell‐like properties in cancer cells [16] are all thought to contribute significantly to radiotherapy resistance. The underlying molecular mechanisms of radiotherapy resistance formation in colorectal cancer remain to be fully elucidated [17]. The establishment of radioresistant colorectal cancer cell lines provides a basis for in‐depth studies of the molecular events and signaling pathways involved in radioresistance.

In this study, we repeatedly irradiated HT29 and HCT116 cells with a conventional dose of 2 Gy per fraction to establish HT29R and HCT116R radioresistant cell lines. We then observed the differences in ionizing radiation effects on these cell lines compared with their parental counterparts, focusing on radiotherapy tolerance, redox status, and DNA damage repair. These radioresistant cell lines serve as a cytological model for further studies on the molecular mechanisms of radiotherapy resistance in colorectal cancer.

## Materials and methods

### Cells culture

Human colorectal cancer cell lines HT29 (catalog no. BFN60800646) and HCT116 (catalog no. BFN607200665) were obtained from the Shanghai Cell Bank of the Chinese Academy of Sciences. The cells were verified by mycoplasma detection and Short Tandem Repeat (STR) analysis. For culturing, the cells were maintained in RPMI‐1640 medium (Gibco, Grand Island, NY, USA) supplemented with 10% FBS (Biological Industries, Kibbutz Beit Haemek, Israel) and penicillin/streptomycin (100 U·mL^−1^) in a cell incubator at 37 °C with 5% CO_2_.

### Reagents and antibodies

Glutathione (GSH) assay kit (catalog no. A006‐2‐1) was obtained from Nanjing Jiancheng Bioengineering Institute. Antibodies against γH2AX (catalog no. YP‐Ab‐01107) and MRE11 (catalog no. YP‐Ab‐00453) were purchased from Youpin Biotechnology Co., Ltd (Hongshi, China). Antibodies against Ki‐67 (catalog no. AF0198), 4‐HNE (catalog no. GTX01087), and fluorescent secondary antibody (catalog no. SA00013‐3) were obtained from Genetex Biotechnology (Alton Pkwy Irvine, CA, USA). Antibodies against Tubulin (catalog no. 66031‐1‐Ig), Ku80 (catalog no. 66546‐1‐Ig), Anti‐mouse IgG, HRP‐linked antibody (catalog no. SA00001‐1), and Anti‐rabbit IgG, HRP‐linked antibody (catalog no. SA00001‐2) were purchased from Proteintech Biotechnology (Wuhan, China).

### Animal preparation and ethics statement

Six‐ to eight‐week‐old male BALB/c nude mice were purchased from Beijing Sibeifu Biotechnology Co., Ltd (Beijing, China). The mice were housed in pathogen‐free facilities maintained at a controlled temperature of 22–23 °C with *ad libitum* access to food and water. All animal procedures were conducted according to the guidelines outlined in the Guide for the Care and Use of Laboratory Animals, and ethics approval (Approval Document No. (2022)‐2‐45) was obtained from the Animal Care and Use Committee of The First People's Hospital of Zunyi (The Third Affiliated Hospital of Zunyi Medical University).

### Ionizing radiation

Single‐cell suspensions were subcultured in six‐well plates. A 6 MV flattening filter (FF) photon beam produced by a linear accelerator (Elekta Precise VMAT, Stockholm, Switzerland) was used for irradiation at a dose rate of 303 cGy·min^−1^. The distance between the source and the skin was 100 cm. Cells were subjected to 2 Gy per irradiation (total 50 Gy) to establish the radiotherapy‐resistant cell lines. In follow‐up radiosensitivity studies, cells were irradiated by gradient fraction doses of 2, 4, or 6 Gy using a variable collimator.

### CCK‐8 assays

For the CCK8 assay, 5 × 10^3^ cells were seeded per well in a 96‐well plate. After allowing the cells to adhere, they were irradiated with doses ranging from 2 to 6 Gy and then incubated for an additional 72 h. Alternatively, cells were irradiated with 4 Gy and incubated for 24–72 h before the CCK8 assay. To perform the assay, 100 μL of medium containing 10% CCK‐8 solution was added to each well. The cells were incubated at 37 °C for 2 h. Blank wells were also included. Absorbance at 450 nm was measured using a multifunction enzyme labeler to determine cell viability.

### Colony formation assay

To perform the colony formation assay, cells were prepared as a single‐cell suspension and seeded into 60‐mm culture dishes at densities of 500, 1000, 2000, and 4000 cells per dish. After allowing the cells to adhere for 12 h, the cells were irradiated with doses of 0 Gy, 2 Gy, 4 Gy, and 6 Gy. The irradiated cells were then incubated in a 37 °C, 5% CO_2_ incubator for 14 days, with complete medium changes every 3 days. After incubation, the cells were stained with 1% crystal violet, colonies were observed and counted in each dish. Survival curves were fitted using the graphpad prism 8 software (San Diego, CA, USA) with both the multitarget single‐hit model ($S=1-{\left(1-e^{-D/D0}\right)}^{N}$) and the linear‐quadratic model $\left(S=e^{\left(-\alpha D-{\beta D}^{2}\right)}\right)$ to calculate radiation sensitivity parameters, including *D*0, *Dq*, *N*, SF2, α, β, and α/β.

### Dihydroethidium (DHE) staining

Cells were inoculated into 24‐well plates and allowed to adhere to the wall. Then, according to the experimental groups, they were irradiated with 4 Gy X‐ray and cultured for an additional 24 h. The plates were removed, and the culture medium was discarded. Subsequently, the cells were incubated with a 10 mmol·L^−1^ working solution of DHE (Shanghai Yi Sheng Biotechnology Co., Ltd., Shanghai, China) for 1 h at room temperature. DAPI (Solarbio, Beijing, China) and an antifluorescence attenuating sealing agent were then added. Finally, the cells were observed using a fluorescence microscope (Olympus, Tokyo, Japan).

### Glutathione (GSH) determination assay

Following the manufacturer's instructions for the GSH assay kit, we first measured the protein concentration of the homogenate samples using the BCA method. We then quantified the GSH content in the colorectal cancer cells at 405 nm using a Multi‐function Enzyme Labeler (Berten Instruments, Montgomery, TX, USA). The following formula was used to calculate the GSH content: GSH content of the cells (μmol·gprot^−1^) = (OD value of the assayed wells − OD value of the blank wells)/(OD value of the standard wells − OD value of the blank wells) × concentration of the standard tubes (20 μmol·L^−1^) × dilution times of the sample pretreatment (twofold) ÷ Protein concentration of homogenate to be assayed (gprot·L^−1^).

### Immunofluorescence assay

Cells were seeded onto glass coverslips and allowed to adhere. After irradiation with 4 Gy, the cells were fixed with 4% paraformaldehyde for 15 min at 0, 1, and 12 h postradiation (hpi). Following fixation, the plates were washed with PBS buffer. Cells were then permeabilized with 0.5% Triton X‐100 for 20 min, blocked with 5% BSA in PBS for 1 h at room temperature, and incubated with primary antibody γH2AX (dilution 1 : 200) at 4 °C overnight. After three washes with PBST, the cells were incubated with fluorescent secondary antibody (dilution 1 : 200) for 1 h at room temperature. The cells were washed again with PBST, stained with DAPI, and examined using a fluorescence microscope.

### Western blotting analysis

HT29, HCT116, and their corresponding radioresistant cells (HT29R, HCT116R) were cultured in six‐well plates. Once the cells reached around 80% confluence, they were collected for protein extraction. Whole‐cell lysates were prepared, and total protein was quantified using the BCA method. The protein samples were then separated by electrophoresis on 10% SDS/PAGE gels and transferred to PVDF membranes (Millipore, Billerica, MA, USA). The membranes were blocked with a protein‐free rapid containment solution (Epizyme, Shanghai, China) for 30 min. Primary antibodies against MRE11 (dilution 1 : 1000), Tubulin (dilution 1 : 40 000), and Ku80 (dilution 1 : 10 000) were incubated with the membranes at 4 °C overnight. After washing with TBST, the membranes were incubated with an HRP‐conjugated secondary antibody (dilution 1 : 5000). Protein bands were detected using the Omni‐ECL Ultra Sensitive Chemiluminescence Detection Kit (Epizyme) and visualized using a Gel Imaging System (Bio‐Rad ChemiDoc MP, Hercules, CA, USA).

### Tumor xenograft model

HT29 and HT29R cells were suspended at 1 × 10^6^ cells in 100 μL PBS and injected subcutaneously into the lateral aspect of the right thigh muscle of 6‐ to 8‐week‐old BALB/c nude mice. The mice were randomly divided into four groups (*n* = 5 per group): HT29, HT29R, HT29 + 10 Gy irradiation, and HT29R + 10 Gy irradiation. Tumor size was measured using calipers, and volume was calculated using the formula: volume (mm^3^) = (length × width^2^)/2. When tumor volume reached 150–200 mm^3^, tumor‐bearing mice were irradiated with a single dose of 10 Gy using an electron beam (collimator 10 × 10 mm, dose rate 303 cGy·min^−1^). The irradiated mice were observed for about 2 weeks; the allowed maximal tumor size/burden (2 cm diameter) was not exceeded. At the study endpoint, the mice were humanely sacrificed via cervical dislocation, and the tumors were removed, and their weights were measured. Then, tumors were embedded in paraffin and sliced for further assay.

### Immunohistochemistry (IHC) assay

Mouse xenograft tumor tissues were embedded in paraffin, deparaffinized, blocked, and then incubated with Ki‐67 (dilution 1 : 200) and 4‐HNE (dilution 1 : 300) for 3 h at room temperature. Subsequently, the tissue sections were incubated with secondary antibodies (catalog no: JHA01) for 1 h at room temperature. Staining was performed using the Universal DAB Chromogenic Kit (catalog no.: ZLI‐9018) after washing. Finally, images were captured following staining and mounting of the slides. Each tumor tissue slide was photographed randomly in at least five fields using a microscope (Nikon Eelipse CIS, Tokyo, Japan) and the relative average optical densities of immunohistochemical staining images were analyzed with the imagej software (Silver Springs, MD, USA).

### Statistical analysis

Data were presented as mean ± standard deviation (SD) and were analyzed using the graphpad prism 8.0 software (San Diego, CA, USA). Two‐way ANOVA was used to compare data between multiple groups, followed by Tukey's multiple comparisons test. Statistical significance was set at *P* < 0.05 (*P < 0.05, **P < 0.01).

## Results

### Morphological changes of colorectal cancer cells after irradiation

**Fig. 1:**
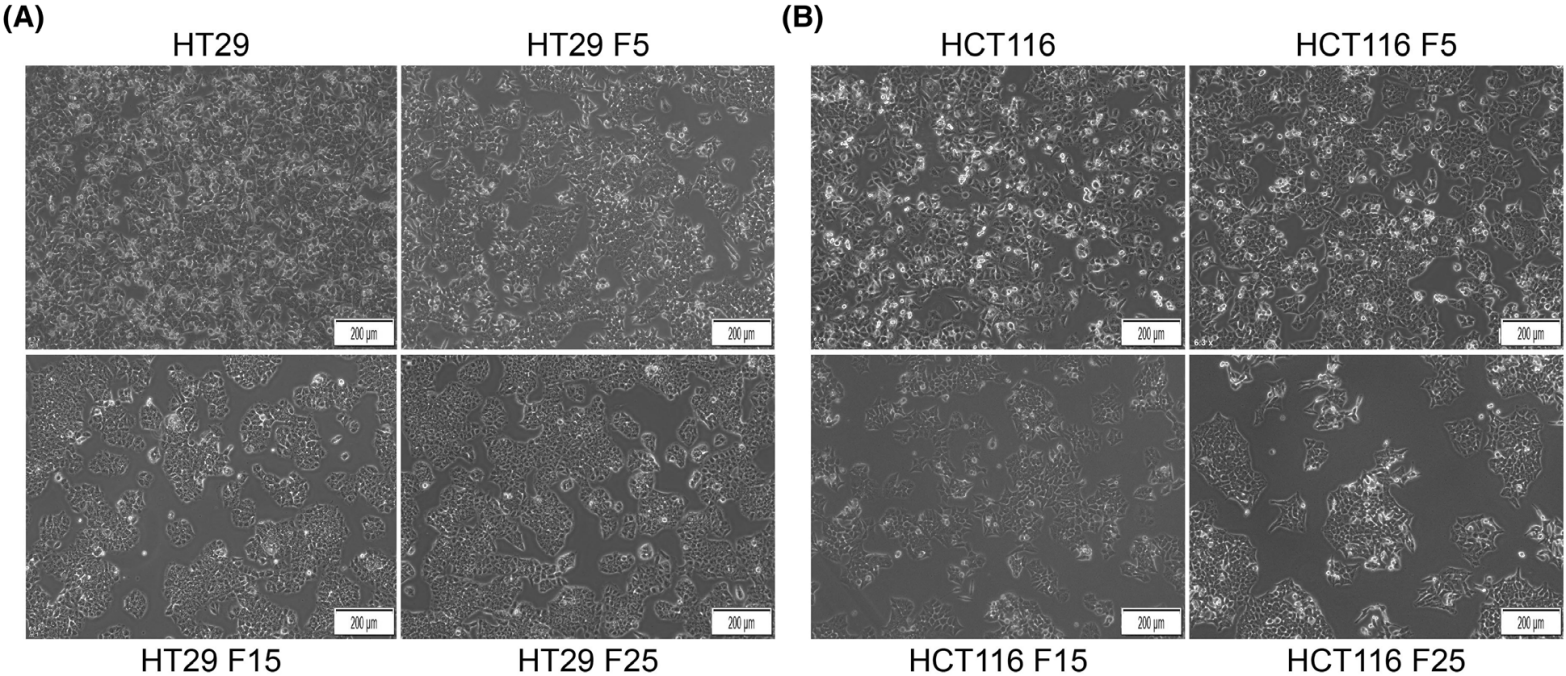
Morphological characteristics of colorectal cancer cells under different fractions of irradiation. Colorectal cancer HT29 (A) and HCT116 (B) cells were irradiated by 2 Gy X‐rays at 5 fractions (F5), 15 fractions (F15) and 25 fractions (F25); morphological characteristics of cells were observed by inverted phase contrast microscopy (×100). The results were derived from three independent experiments. Scale bar: 200 μm.

To explore the characteristics of radiotherapy‐induced radioresistance in CRC, radioresistant cell models were established using HT29 and HCT116 cell lines. Cells were cultured in T25 culture flasks and irradiated with an equal dose of 2 Gy per fraction (25 fractions, total dose 50 Gy) using an Elekta Precise VMAT linear accelerator. Inverted phase contrast microscopy was used to observe the morphological changes of parental and irradiated cells after receiving ionizing radiation at 5 fractions (F5), 15 fractions (F15), and 25 fractions (F25). We observed that the irradiated cancer cells gradually transformed from their original polygonal and irregular shape to round and oval. Some cells fused into patches, showing a paving‐stone‐like arrangement, and clusters exhibiting clumped and adherent growth patterns (Fig. 1A,B).

### Validation of the radioresistance of irradiated colorectal cancer cells *in vitro*

**Fig. 2:**
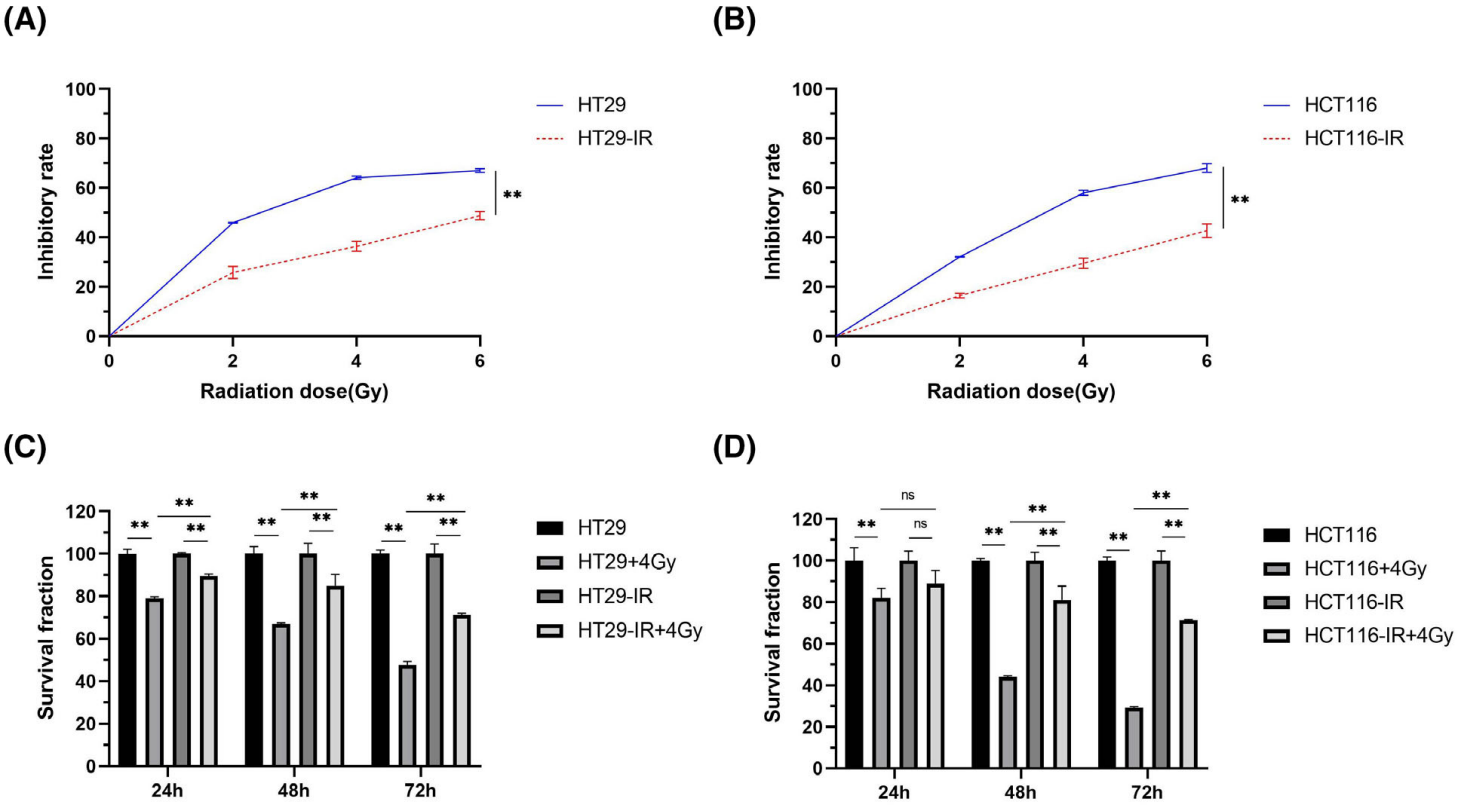
Effect of irradiation on the viability of colorectal cancer cells. After 72 h of irradiation with 0–6 Gy, the cell viability of parental HT29 and irradiated HT29 (HT29‐IR) cells (A) or parental HCT116 and irradiated HCT116 (HCT116‐IR) cells (B) was detected by CCK8 assay, and the survival and inhibition rates were calculated. When cells were irradiated at 4 Gy, the survival rate of HT29/HT29‐IR (C) and HCT116/HCT116‐IR cells (D) was assayed at 24~72 h. The results were derived from three independent experiments, and the values were presented as mean ± SD. Statistical analysis utilized two‐way ANOVA and Tukey's multiple comparisons test (ns: non‐significant, *P* > 0.05, **P < 0.01).

The cancer cells that cumulatively received a total dose of 50 Gy were designated HT29‐IR and HCT116‐IR cells, respectively. The CCK‐8 assay was used to assess the survival of these cells and their parental counterparts (HT29 and HCT116) following exposure to different radiation doses (2–6 Gy) for 72 h. The results showed significantly higher radiation‐induced inhibition of parental cell survival compared with HT29‐IR and HCT116‐IR cells (Fig. 2A,B). Furthermore, when parental HT29 and HCT116 cells, as well as the radioresistant cells, were re‐irradiated with a dose of 4 Gy for varying time points (24–72 h), the survival of both cell types decreased significantly with increasing incubation time. However, the survival rate of both HT29‐IR and HCT116‐IR cells remained significantly higher than that of their corresponding parental cells (Fig. 2C,D). These findings indicate that HT29‐IR and HCT116‐IR cells developed resistance to radiation.

**Fig. 3:**
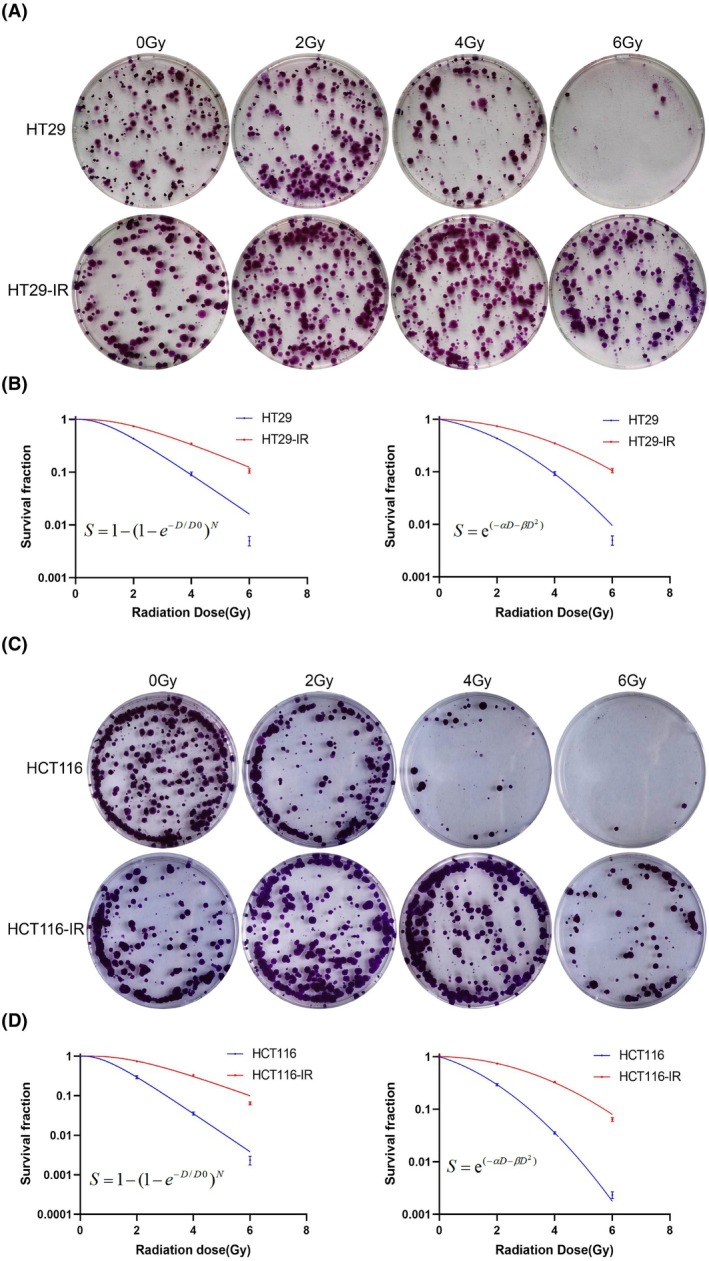
Effects of different radiation doses on the proliferative capacity of parental and irradiated colorectal cancer cells. Following irradiation and incubation for 14 days, colony formation assays were performed to assess the growth abilities of (A) HT29 and HT29‐IR cells and (C) HCT116 and HCT116‐IR cells. (B) Survival curves of HT29 and HT29‐IR cells and (D) HCT116 and HCT116‐IR cells were fitted using the multitarget click model and linear‐quadratic model. Data are presented as mean ± SD from three independent experiments. Statistical analysis was performed using two‐way ANOVA with Tukey's multiple comparisons test.

To further assess the resistance of irradiated colorectal cancer cells, we assessed the colony formation ability of parental and irradiated cells. Following irradiation with 2–6 Gy X‐rays, the number of colonies formed by both HT29‐IR and HCT116‐IR cells was significantly higher than HT29 and HCT116 parental cells (Fig. 3A,C). The dose–survival curves of parental and irradiated cells were fitted using the multitarget single‐hit model and the linear‐quadratic model, respectively (Fig. 3B,D). The calculated radiobiological parameters (Table 1) indicated that both HT29‐IR and HCT116‐IR cells acquired significant resistance to radiotherapy, becoming radioresistant (HT29R and HCT116R) cells.

**Table 1:** Parameters of dose–survival curves for colorectal cancer parental and radiotherapy‐resistant cells. *D*0, final slope; *Dq*, quasi‐threshold dose; *N*, extrapolation number; SF2, survival fraction at 2 Gy irradiation; α, single‐hit biological effectiveness factor; β, multitarget biological effectiveness factor.

Cell line	Single‐hit multitarget model				Linear‐quadratic model		
	*D*0	*Dq*	*N*	SF2	α	β	α/β
HT29	1.16 ± 0.03	1.23 ± 0.01	2.89 ± 0.08	0.43 ± 0.01	0.24 ± 0.01	0.09 ± 0.01	2.73 ± 0.10
HT29‐IR	1.86 ± 0.10^a^	2.20 ± 0.11^a^	3.26 ± 0.42	0.74 ± 0.02^a^	0.04 ± 0.01^a^	0.06 ± 0.01^a^	0.74 ± 0.25^a^
HCT116	0.90 ± 0.02	1.00 ± 0.09	3.03 ± 0.39	0.29 ± 0.02	0.40 ± 0.06	0.11 ± 0.01	3.55 ± 0.90
HCT116‐IR	1.65 ± 0.06^b^	2.24 ± 0.06^b^	3.88 ± 0.31	0.74 ± 0.01^b^	0.03 ± 0.01^b^	0.07 ± 0.01^b^	0.19 ± 0.02^b^

^a^ Compared to HT29, *P* < 0.01

^b^ Compared to HCT116, *P* < 0.01.

### *In vivo* validation of the radiosensitivity of CRC radioresistant cells

**Fig. 4:**
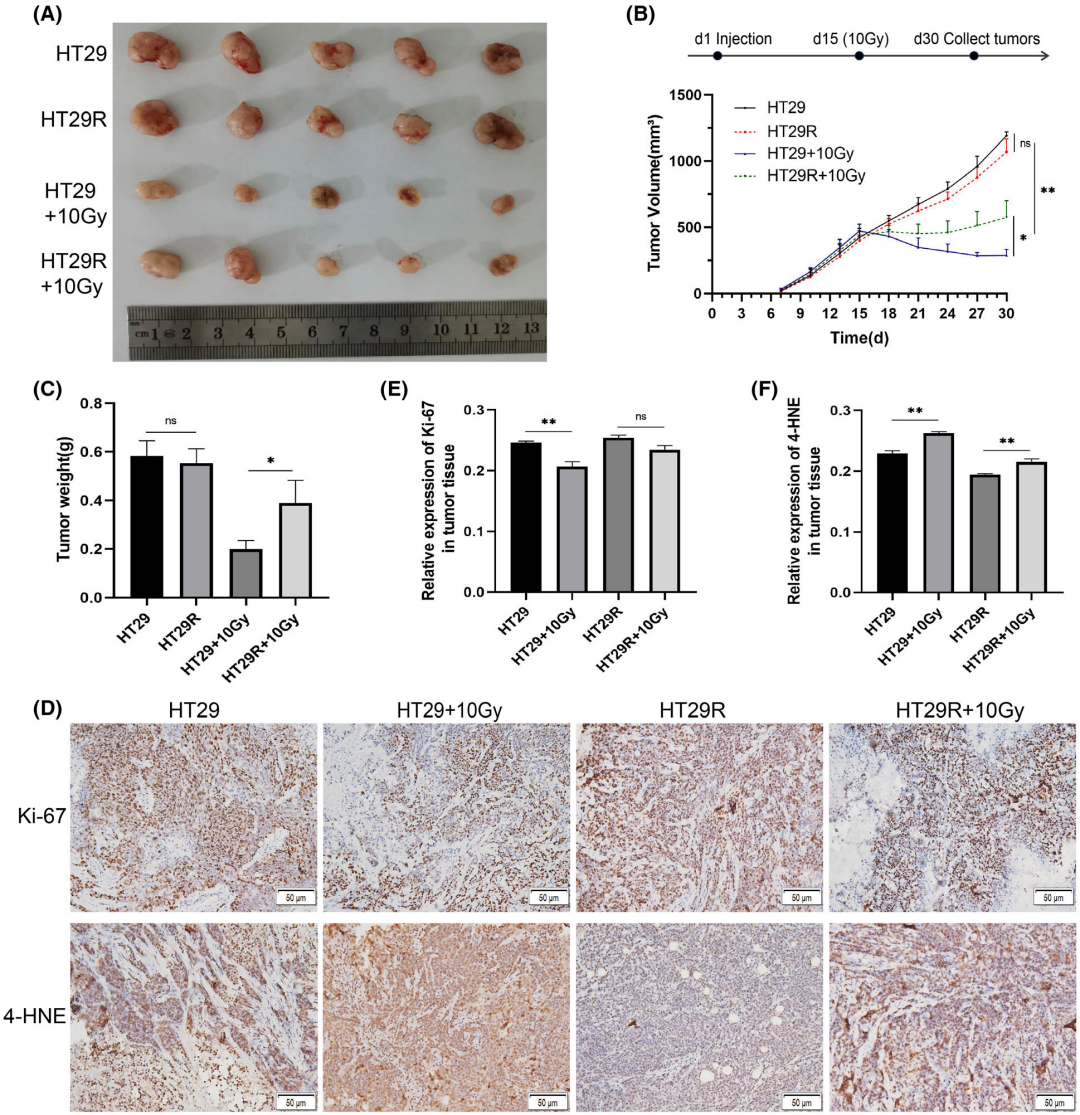
Validation of sensitivity of colorectal cancer cells to ionizing radiation by tumor formation experiments in mice. Nude‐mouse transplanted tumor model was established by subcutaneously implanting HT29 parental and radioresistant cells to assay the radiation tolerance of cancer cells, and the gross appearance of tumors (A), tumor volume (B) and tumor weights (C) in each group were assayed; (D) Immunohistochemical staining for Ki67 and 4‐HNE in tumor tissue of mice. Quantitative analysis of immunohistochemical staining for Ki67 and 4‐HNE in tumor tissue of mice (E, F). The experiments were repeated three times independently, and the results are shown as mean ± SD. Statistical analysis utilized two‐way ANOVA and Tukey's multiple comparisons test (ns: nonsignificant, *P* > 0.05, *P < 0.05, **P < 0.01). Scale bar: 50 μm.

To further validate radioresistance *in vivo*, we employed HT29 and HT29R cells in nude mouse tumorigenicity experiments. We evaluated tumor sensitivity to irradiation after subcutaneous tumor formation. As shown in Fig. 4A–C, no significant differences in tumor volume or weight were observed between the HT29 and HT29R nonirradiated groups. However, following 10 Gy irradiation, tumor growth in the HT29 cell group was significantly inhibited. Tumor volume and weight were reduced to (288.54 ± 90.18) mm^3^ and (0.198 ± 0.07) g, respectively. In contrast, the HT29R irradiated group displayed significantly higher tumor volume [(575.43 ± 191.81) mm^3^] and weight [(0.39 ± 0.13) g] than the HT29 unirradiated group (*P* < 0.05). These findings indicated that subcutaneous tumors derived from HT29R cells exhibited significantly greater resistance to radiotherapy compared to tumors derived from HT29 parental cells.

Immunohistochemistry was performed to detect changes in the expression of Ki‐67 (proliferation marker) and 4‐HNE (oxidative stress marker) within subcutaneous tumor tissues. Following 10 Gy irradiation, Ki‐67 expression in HT29R tumor tissues was significantly higher than that in the control group. Conversely, 4‐HNE expression in HT29R tumors was significantly lower than the HT29 parental cell tumors (Fig. 4D–F). These findings suggest that ionizing radiation effectively increased oxidative damage and inhibited the proliferative activity of cancer cells in the parental tumor group. In contrast, the transplanted HT29R tumors displayed relative tolerance to ionizing radiation, with cancer cells exhibiting a higher proliferative potential and a stronger ability to resist oxidative damage.

### Expression of DNA damage and repair‐related proteins in CRC cells

**Fig. 5:**
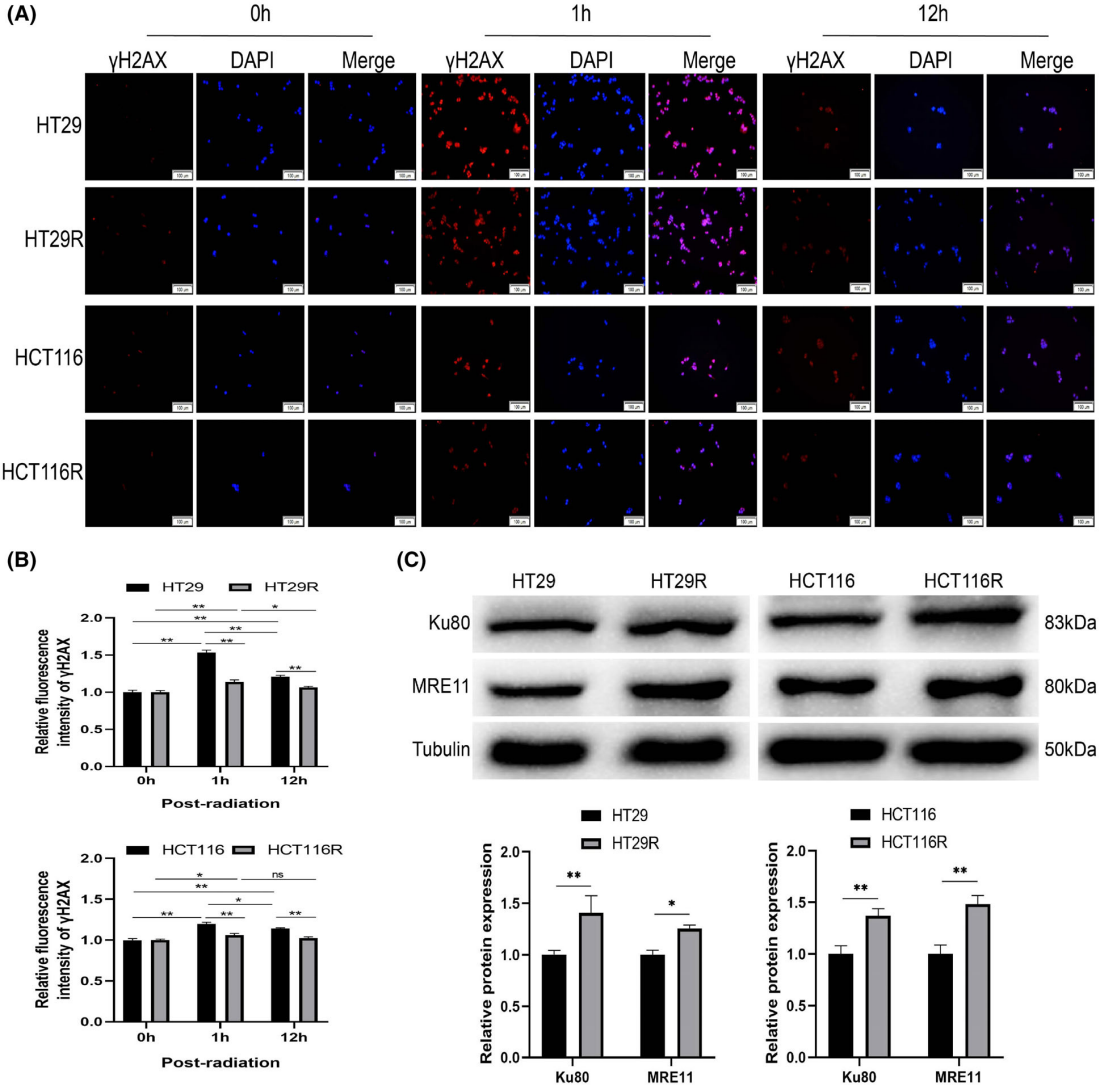
Assessment of DNA damage and repair capacity in colorectal cancer cells. After 4Gy irradiation, immunofluorescence staining was used to detect the expression of γH2AX in HT29 or HCT116 parental and radioresistant cells at 0, 1, and 12 h (A); and quantitative analysis of γH2AX expression in cells (B). (C) The expression of Ku80 and MRE11 in cells was detected by western blot. The results were derived from three independent experiments, and the values were presented as mean ± SD. Statistical analysis utilized two‐way ANOVA, Tukey's multiple comparisons test (*P < 0.05, **P < 0.01). Scale bar: 100 μm.

Colorectal cancer cells exhibit a stress response and self‐adaptation process after undergoing radiotherapy. This phenomenon is rooted in the interplay between intracellular DNA damage caused by ionizing radiation and the subsequent DNA repair mechanisms. To investigate these processes, we employed immunofluorescence to detect γH2AX expression in HT29/HCT116 and HT29R/HCT116R cells following irradiation. Our results revealed a robust γH2AX fluorescence signal in HT29 and HCT116 cells at 1 h postirradiation, persisting up to 12 h. While γH2AX expression was also observed in resistant cells, it was markedly weaker than their parental counterparts, particularly at the 12‐h time point (Fig. 5A,B). Western blot analysis further demonstrated a significant upregulation of MRE11 and Ku80 proteins in resistant cells compared with parental cells. MRE11 plays a key role in DNA homologous recombination repair, while Ku80 is involved in nonhomologous end‐joining repair (Fig. 5C). These findings suggest that DNA damage induced by ionizing radiation is less pronounced in colorectal cancer‐resistant cells, potentially due to enhanced DNA repair capacity.

### Generation of ROS and GSH consumption in CRC parental and radioresistant cells

**Fig. 6:**
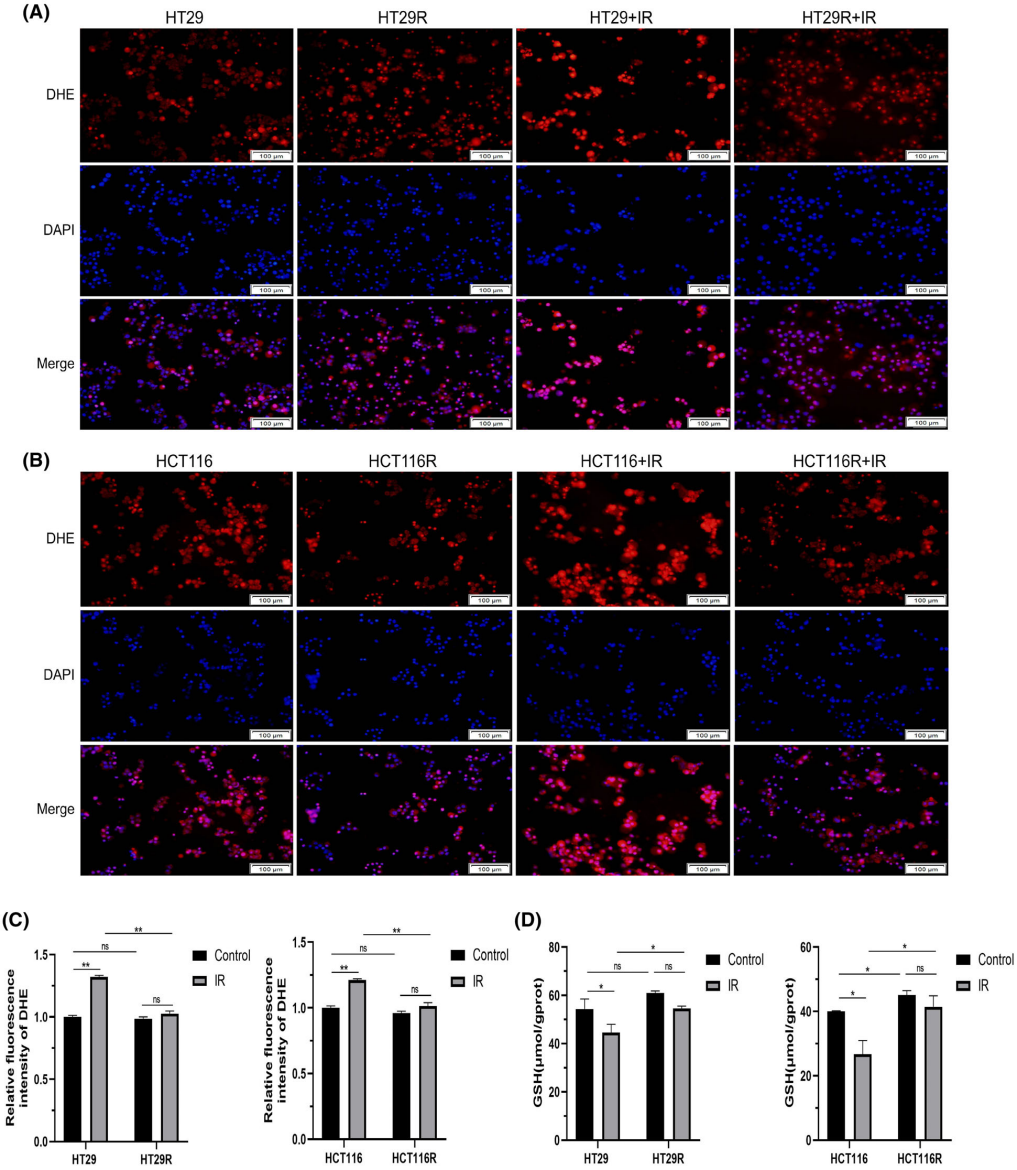
Effects of ionizing radiation on ROS generation and GSH depletion in colorectal cancer cells. After 48 h of irradiation with 4Gy, DHE staining was used to observe ROS generation in HT29/HT29R (A) or HCT116/HCT116R (B) cells, and the results of DHE staining were quantitatively analyzed (C); the levels of GSH in HT29/HT29R or HCT116/HCT116R cells were also detected (D). The results were derived from three independent experiments, and the values were presented as mean ± SD. Statistical analysis utilized two‐way ANOVA, Tukey's multiple comparisons test (ns: nonsignificant, *P* > 0.05, *P < 0.05, **P < 0.01). Scale bar: 100 μm.

To further understand the changes in ROS levels in colorectal cancer parental and resistant cells after radiotherapy, we used the fluorescent probe DHE staining to observe the effects of irradiation (4 Gy) on intracellular ROS. We found that DHE staining in parental cells was significantly enhanced after ionizing radiation, while DHE fluorescence intensity in postirradiated HT29R and HCT116R cells only showed a slight increase compared with control cells (Fig. 6A–C). GSH, the main intracellular reducing substance, directly affects the intracellular redox level. To investigate the intracellular antioxidant damage capacity of colorectal cancer parental and radiotherapy‐resistant cells, we measured changes in GSH content. The results showed that after simultaneous irradiation with 4 Gy X‐rays, the content of GSH in HT29 and HCT116 parental cells decreased from (54.38 ± 3.36) μmol·gprot^−1^ to (44.60 ± 2.78) μmol·gprot^−1^ and (40.07 ± 0.25) μmol·gprot^−1^ to (26.71 ± 4.28) μmol·gprot^−1^, respectively (both *P* < 0.05). In contrast, GSH levels in HT29R cells ((60.93 ± 0.63) μmol·gprot^−1^ vs. (52.41 ± 3.13) μmol·gprot^−1^) and HCT116R cells ((45.11 ± 1.94) μmol·gprot^−1^ vs. (41.49 ± 4.78) μmol·gprot^−1^) showed no significant changes before and after undergoing radiotherapy (Fig. 6D).

## Discussion

Radiotherapy is a cornerstone therapy for CRC, alongside surgery and medication [18]. However, the emergence of radiotherapy resistance significantly hinders treatment success, posing a substantial challenge for both clinicians and patients [13, 19]. Overcoming cancer cell radioresistance, thereby enhancing tumor radiosensitivity and ultimately improving radiotherapy efficacy, represents a major area of ongoing research in CRC management [20, 21].

*In vitro* establishment of radiotherapy‐resistant cell lines using ionizing radiation offers a valuable tool to elucidate the mechanisms behind resistance, ultimately aiding the discovery of novel molecular targets and therapeutic strategies to overcome it [22, 23]. However, a standardized method for achieving this is lacking. Two main approaches exist: large split irradiation (single dose exceeding 6 Gy) and conventional split irradiation (single dose between 1.8 and 2.0 Gy) [24]. While large split irradiation utilizes fewer treatment sessions with higher single doses, the high percentage of lethal damage initially necessitates a longer recovery period for the cells to regain proliferative capacity. Conversely, conventional split irradiation employs smaller single doses, requiring a larger number of sessions and a longer overall modeling period to achieve a comparable biological effect. This extended time frame, unfortunately, increases the susceptibility of cells to contamination from environmental or operational factors, potentially leading to failed model development.

This study employed a single dose of 2 Gy per irradiation, mimicking the clinical routine of radiotherapy, to deliver a cumulative dose of 50 Gy to HT29 and HCT116 cells. By evaluating changes in growth morphology, *in vitro* proliferation survival, and colony‐forming ability, we confirmed that irradiated cells exhibited increased radiation tolerance compared with their parental counterparts, demonstrating their transformation into radioresistant cells. Furthermore, the radioresistance of HT29R‐derived tumors was established by injecting parental and radioresistant cells subcutaneously into nude mice and observing their response to radiation treatment. In contrast, conventional fractionated radiotherapy, which more closely resembles the daily treatment regimen used in clinical settings, allows for greater intervals between irradiation sessions, potentially facilitating DNA repair in cancer cells. This cyclic pattern of damage‐repair–re‐damage‐rerepair theoretically reflects the gradual adaptation of cancer cells to the radiation environment. Exploring the molecular mechanisms underlying resistance development in this context would provide a deeper understanding of the nature of molecular signaling events triggered by radiation exposure in colorectal cancer.

Studies have shown that cancer cells exhibit significant changes in gene expression profiles before and after radiation exposure. Notably, genes involved in DNA damage repair pathways become more highly expressed after radiation, particularly those sensitive to DNA damage, such as Mre11, Rad50, BRCA1/2, and NBS1 involved in homologous recombination repair, and XRCC4, Ku70/80 involved in nonhomologous end‐joining [25, 26]. This upregulation is often associated with the development of radiotherapy resistance. Consistent with these findings, our study demonstrated that MRE11 and Ku80 proteins were significantly upregulated in HT29R and HCT116R cells compared with their nonirradiated parental cells. These results suggest that DNA repair, the most fundamental and prevalent molecular response of tumor cells to ionizing radiation, serves as the molecular basis for the gradual adaptation and tolerance of cancer cells to radiation [27].

Ionizing radiation generates significant intracellular reactive oxygen species (ROS), leading to oxidative stress and cell death, forming the basis for radiation‐induced cancer cell killing. However, the extent of oxidative damage in colorectal cancer radiotherapy‐resistant cells remains unclear. Our study demonstrates that re‐irradiated resistant cells produce significantly lower ROS levels than parental cells. Additionally, these resistant cells exhibit higher levels of GSH, a crucial intracellular antioxidant that effectively protects cells from oxidative damage [28]. Elevated GSH levels likely contribute to the resistance phenotype by enabling cancer cells to counteract ionizing radiation‐induced apoptosis and ferroptosis [29, 30]. Therefore, the enhanced antioxidant capacity observed in colorectal cancer‐resistant cells likely represents another mechanism underlying radiation resistance. Elucidating the regulatory mechanisms involved in this process could provide novel molecular therapeutic targets to overcome radiotherapy tolerance in colorectal cancer.

Taken together, we successfully established radiotherapy‐resistant colorectal cancer cells (HT29R and HCT116R) by accumulating a total dose of 50 Gy through 25 fractions of 2 Gy each, mimicking a conventional split‐dose radiotherapy regimen. Both *in vitro* and *in vivo* experiments confirmed the significant radioresistance of these cells. Compared with their parental counterparts, HT29R and HCT116R cells exhibited enhanced DNA damage repair capability and antioxidant capacity. These findings establish a reliable cell model for investigating the molecular mechanisms underlying radiotherapy resistance in intestinal cancer. Furthermore, they offer a novel perspective for studying resistance by focusing on oxidative damage homeostasis.

## Conflict of interest

The authors declare no conflict of interest.

## Author contributions

T‐YQ, QD: writing – original draft, visualization, methodology. JL, LF: supervision, software, investigation. J‐JM, RC: software, resources, investigation. CC, P‐FR: validation, methodology, formal analysis. W‐WZ, CL: visualization, data curation, conceptualization. H‐FY: writing – review and editing, funding acquisition, validation, resources.

## Data Availability

The data that support the findings of this study are available from the corresponding author upon reasonable request.
